# Effect of the Icelandic Mutation APP^A673T^ in the Murine APP Gene on Phenotype of Line 66 Tau Mice

**DOI:** 10.3390/biom16010028

**Published:** 2025-12-24

**Authors:** Anne Anschuetz, Lianne Robinson, Miguel Mondesir, Valeria Melis, Bettina Platt, Charles R. Harrington, Gernot Riedel, Karima Schwab

**Affiliations:** 1 School of Medicine, Medical Sciences and Nutrition, University of Aberdeen, Foresterhill, Aberdeen AB25 2ZD, UK; a.anschuetz.21@abdn.ac.uk (A.A.); lianne.strachan@abdn.ac.uk (L.R.); mmondesi@ed.ac.uk (M.M.); v.melis@abdn.ac.uk (V.M.); b.platt@abdn.ac.uk (B.P.); c.harrington@abdn.ac.uk (C.R.H.); karima.schwab@abdn.ac.uk (K.S.); 2 TauRx Therapeutics Ltd., 395 King Street, Aberdeen AB24 5RP, UK

**Keywords:** tau, amyloid-beta, Icelandic mutation, dementia, synaptic proteins, behaviour

## Abstract

The Icelandic mutation in the amyloid precursor protein (APP), APP^A673T^, has been identified in Icelandic and Scandinavian populations and is associated with a significantly lower risk of developing Alzheimer’s disease (AD). The introduction of the human APP^A673T^ form led to a reduction in amyloid β-protein (Aβ) production and tau pathology, but the effect of mouse APP^A673T^ on tau and Aβ pathology is not well studied. We have crossed line 66 (L66) tau transgenic mice that overexpress the P301S aggregation-prone form of tau with C57Bl6/J mice expressing a single-point mutation edited into the murine *APP* gene via CRISPR-Cas gene editing, known as mAPP^A673T^. We have performed ELISA, histopathological, and behavioural analyses of heterozygous male/female L66 and L66 xmAPP^A673T^ crosses at the age of 6 months to investigate the effect of the murine A673T mutation on tau brain pathology and behavioural deficits in these mice. Using immunohistochemistry, we found only a moderate, yet significant, reduction in mAb 7/51-reactive tau for female L66 x mAPP^A673T^ compared to L66 mice. Quantification of tau in soluble/insoluble brain homogenate fractions by ELISA confirmed the lack of overt differences between genotypes, as did our extensive behavioural phenotyping using six different paradigms assessing motor function, olfaction, depression/apathy-like behaviour, as well as exploration and sociability. Therefore, the mAPP^A673T^ mutation has a moderate impact on tau pathology but does not appear to impact motor and neuropsychiatric behaviour in L66 tau transgenic mice.

## 1. Introduction

Dementia, including Alzheimer’s disease (AD), is an incurable disease characterised by a progressive and irreversible decline in cognition leading to disoriented behaviour, altered personality, aggression, agitation, anxiety, difficulties with speech and comprehension, and impaired gait and movement [1]. The decline is related to a loss of neuronal function caused by the formation of neurofibrillary tangles and extracellular, neuritic plaques [2]. These two hallmarks of AD consist mainly of the microtubule-associated protein tau [3,4,5,6,7] with amyloid β-protein (Aβ) present also in neuritic and non-neuritic plaques [8,9,10]. Tau protein was identified as a polymerisation factor for microtubules, which promotes microtubule assembly and provides axonal stabilisation [11]. In addition, tau may also be involved in the regulation of axonal-guided transport through interactions with motor proteins and other binding partners [12]. Amyloid-β is generated through the cleavage of the amyloid precursor protein (APP), a protein that functions in a variety of physiological processes, including modulation of synaptic function, facilitation of neuronal growth and survival, and protection against oxidative stress [13,14].

Early work has shown that deletion of tau leads to altered microtubule organisation and complex motor deficits, including impaired performance on the rotarod and decreased locomotion in the open field test [15,16], while deletion of the amyloid precursor protein has also been associated with hypoactivity and reduced grip strength [17]. By contrast, overexpression of mutated tau in mice led to its accumulation in nerve cell bodies, axons, and dendrites, causing muscle atrophy and dysfunctional sensorimotor performance [18,19,20]. Similarly, some transgenic APP mice also developed neuritic plaques, synaptic loss, and memory impairments [21,22,23]. While the loss of normal function and gain of toxic function of tau and Aβ are clearly implicated in the pathophysiology of AD, for other dementias like frontotemporal dementia (FTD), these pathways do not necessarily interact [24,25,26,27,28,29]. In cultured hippocampal neurons, however, degeneration required the presence of both tau and Aβ-treatment [30], and that reducing endogenous tau levels without altering Aβ prevented behavioural deficits in Aβ-transgenic mice, emphasising that tau may be essential for Aβ-induced neurotoxicity [31]. Additionally, tau deletion in Aβ-transgenic mice has also been shown to reduce plaque burden [32], and in tau transgenic mice, treatment with anti-tau antibodies reduces Aβ levels [33]. This interplay is further complicated by the different aggregation properties of human and mouse Aβ [34], and whether endogenous mouse Aβ alters human Aβ in transgenic models [35].

To further address the interaction between Aβ and tau in the pathogenesis of AD, FTD, and related disorders, we have crossed mice expressing murine *APP* with the Icelandic mutation, mAPP^A673T^, with line 66 (L66) tau transgenic mice. L66 mice overexpress the P301S aggregation-prone form of tau and are characterised by early-onset tau pathology, and subsequent sensorimotor and motor dysfunction and nest-building deficits [36,37,38,39,40]. In humans, the P301S mutation is associated with FTD with Parkinsonism [41]. APP^A673T^ mice harbour the Icelandic mutation in the murine *APP* gene generated by CRISPR-Cas gene-editing technology. This mutation, first identified in elderly Icelandic and later in different Scandinavian populations, significantly reduces carriers’ risk of developing AD [42,43]. The protective effect of A673T is believed to be primarily achieved through reduced β-cleavage of APP [44,45,46]. The human A673T mutation proved protective against Aβ pathology in Aβ-transgenic mice [47] but failed to do so in an Aβ inoculation model, where a decrease in tau pathology was found instead [48]. However, the effect of the murine A673T mutation on tau pathology/aggregation in the absence of human Aβ overexpression remains unknown. In this exploratory study, heterozygous L66 x mAPP^A673T^ crosses were analysed both behaviourally and histopathologically relative to L66 tau transgenic mice. Multiple secondary endpoints included determination of synaptic proteins and behavioural phenotypes. We assumed that, if protective, the Icelandic mutation should reduce tau levels in L66 x mAPP^A673T^ relative to L66 mice.

## 2. Materials and Methods

### 2.1. Study Design

The study was exploratory. The primary read-out was tau pathology and whether it is reduced in L66 x mAPP^A673T^ compared to L66. No power calculations were performed a priori, but the group sample size was based on experience and expectations from previous experiments performed on L66 mice [36,37,38,39,40]. The body weight of mice was determined prior to the start of the experiment and once per week during the experimentation period. Welfare recommendations suggested that any mouse with a body weight decline ≥ 15% be excluded from the study, but this did not occur, and no mouse was excluded during the in vivo phase of the study. A randomisation sequence for behavioural testing was generated using the random function in Excel (Microsoft Office) to generate 2 cohorts balanced for sex and genotype. Experimenters and caretakers were blinded to the genotype of the mice during group allocation, behavioural assessment, data acquisition, and primary data analyses. A different investigator performed statistical analyses for behavioural data and was not blinded to any study details. Following tissue collection, an independent experimenter, also blinded to the genotype of mice, performed immunohistochemistry, ELISA assays, and all statistical analyses relating to these measurements.

### 2.2. Animals

All animal experiments were performed in accordance with the European Communities Council Directive (63/2010/EU) with local ethical approval under the UK Animals (Scientific Procedures) Act (1986) and its amended regulations (2012), and under the project licence number PP2213334 compliant with the ARRIVE guidelines 2.0 [49].

Mice were bred commercially in positive-pressure isolators (Charles River, Margate, UK). Homozygous tau transgenic line 66 mice, expressing the full-length human tau transgene htau40 (amino acids 1-441) with two mutations in position 301 (P301S) and 335 (G335D) [37]. Mice were bred on a white NMRI background strain. Mice harbouring the Icelandic mutation were generated by CRISPR-Cas gene-editing technology with a nucleotide modification of the murine *APP* gene, changing amino acid 673 from alanine to threonine, on a black C57Bl6/J background at Jax laboratories (Bar Harbor, ME, USA), termed mAPP^A673T^ mice. Screening for potential off-target sites confirmed 4 low-frequency targets (score < 3.5; for comparison, A673T score is 100) with unlikely consequences for the phenotype (loci: chromosome 18: +538,447,025; chr16: −43,029,129; chr4: +152,414,459; chr13: −4,178,209). These were not confirmed. Here, crosses were bred from homozygous L66 (male or female) with heterozygous mAPP^A673T^ (male or female) mice, and the resulting agouti-coloured offsprings were all heterozygous for the L66 htau40 transgene, but ear biopsies were genotyped for the A673T mutation in the *APP* gene by Transnetyx Inc. (Cordova, AK, USA). These were either mA673T positive or wild type. Fifty-nine 6-month-old mice were included in the study (Table 1). Experimental mice were delivered by truck to the animal facilities at the University of Aberdeen, Scotland, one month prior to the start of the experiments. Mice were kept in sex-specific litters ≥ 2 in stock box open housing under constant environmental conditions (20–22 °C temperature, 50–65% humidity, an air exchange rate of 17–20 changes per hour, and a 12 h light/dark cycle with lights turned on at 7 am with simulated sunrise/sunset) and ad libitum chow (Special Diet Services, Witham, UK) and water throughout. Mice were also provided with corncob bedding, paper strips, and cardboard tubes (DBM Scotland Ltd, Broxburn, UK) as enrichment throughout the experiment. One week before and during behavioural testing, all animals were singly housed to harmonise holding conditions throughout the in-live phase of the experiment. Behavioural testing commenced with motor coordination on the rotarod followed by home cage observations in the PhenoTyper, nest building in the home cage, sucrose preference test, buried cookie test, and social interaction (for details, see below). Mice were maintained singly housed in Macrolon type III cages in the same holding room, except when they were transferred to specialised testing rooms for behavioural assessments or for euthanasia and sacrifice. Mice were given ample time to recover between experiments (>3 days) and 30 min to acclimate to behavioural rooms before any procedure took place.

### 2.3. Behavioural Testing

Behavioural testing was conducted using a battery of tests which consisted of assessments of motoric (Rotarod), exploratory (home cage activity analysis in the PhenoTyper), social interaction, the buried cookie test, and apathy-like/anhedonic behaviours (nest building and sucrose preference tests) performed over four weeks. The age of 6 months was chosen because at this age L66 mice have developed extensive tau pathology in the brain that leads to behavioural dysfunction such as in the rotarod and the nest building test [36,37,38,39,40].

#### 2.3.1. Rotarod

To assess motor function, a four-lane rotarod system was used (Model 33700-R/A; TSE Technical & Scientific Equipment GmbH, Bad Homburg, Germany). Animals were tested for 4 trials per day over 3 consecutive days with inter-trial intervals of 2–3 min during which the apparatus was cleaned with 70% ethanol. Four mice were tested simultaneously, with the run sequence randomised and counterbalanced for time of testing and rod location using a Latin square design. At the start of each trial, the mouse was placed on a slowly rotating rod (1 rpm (rotation per minute)) with its head facing against the rotation. The trial was started once all animals were in position and the rotation speed of the rod was accelerated from 1 rpm to 45 rpm over a period of 5 min. The trial was ended when the animal fell off the rod or when the maximum trial time was reached. The time spent on the rod (sec) for each of the 12 trials (T1–T12) was extracted as the primary read-out and used for analyses.

#### 2.3.2. PhenoTyper

Following the rotarod test, locomotor activity and circadian activity patterns were monitored using the PhenoTyper home cage observation system (Noldus Information Technology, Wageningen, The Netherlands). The cages consisted of clear Perspex walls and opaque floors (30 cm × 30 cm × 35 cm) and contained a food hopper and water bottle, allowing free access to food and water during testing. The cages were filled with sawdust and the top unit of each cage contained a built-in digital infrared sensitive video camera and infrared lighting sources for video tracking. The infrared sources enabled continuous behavioural recordings in both dark and light periods. Animals were individually placed in the PhenoTyper cages with environmental conditions identical to those of the holding rooms. The animals were housed in PhenoTypers for 7 consecutive days and were given two days of habituation followed by assessment of circadian activity from days 3–7. The behaviour of the animals was recorded using the video-tracking software EthoVision XT (Version 16, Noldus Information Technology) by background subtraction at 25 Hz. Habituation to the novel environment was measured by analysing the distance moved during the initial 3 h period following placement in the PhenoTyper with data extracted in 10 min time bins. During the experimental days, the distance travelled was extracted and analysed in 1 h time bins to determine the exploratory and circadian activity of the mice. Thirty mice were tested simultaneously.

#### 2.3.3. Nest Building Test

During single housing in Macrolon Type III cages (Tecniplast, Milan, Italy), a cotton nestlet (50 mm × 50 mm square pressed cotton, DBM Scotland Ltd., UK) was positioned in the cage prior to the start of the dark cycle and the nest-building ability of the mice was visually scored after a period of 16 h (Day 1) and 48 h (Day 2). The scoring was performed by three independent researchers who were blind to the genotype of the mice using the scoring system developed by Deacon [50] and recently validated by Robinson et al. [38]. The scoring system utilised a 5-point scale determined by the completeness of the nest. A score of 1 was assigned if the nestlet remained predominantly untouched. A partially torn-up nestlet was given a score of 2, whilst an almost entirely shredded nestlet but no clear nest location scored 3. Once the nestlet was entirely shredded and a nest area was evident, a score of 4 was assigned for a flat nest with a maximum score of 5 only given when the nest resembled a crater with walls higher than the height of the mouse. The scores of the 3 researchers were averaged for each mouse and used for statistical analysis.

#### 2.3.4. Sucrose Preference

For the sucrose preference test, animals were housed in Perspex cages (54 cm × 50 cm × 37 cm) (Ugo Basile, Comero, Italy). Each cage was filled with corncob bedding and equipped with two drinking bottles and a food hopper. Two days following nest building, the animals were individually placed into sucrose test cages for two days of habituation. Subsequently, water in one bottle was exchanged for 1% sucrose solution and the weights of the two bottles were recorded. The position of the sucrose bottle was counterbalanced (left or right) for animals/groups. Both bottles were weighed after 24 h and the position of the sucrose bottle alternated to avoid any spatial preference. The weights of the two bottles were recorded again following a further 24h, after which the animals were returned to their home cages. Water and sucrose consumption for each animal was averaged for the 48-h period and sucrose preference was determined as the amount of sucrose solution consumed divided by the total intake of fluid multiplied by 100. To control for possible water/sucrose leakages from the bottles, small bags were attached to the bottle holders close to the spouts and used to collect any fluid, which was then subtracted from the bottle weights. Upon completion of the 48-h test, animals were returned to their Macrolon home cages.

#### 2.3.5. Buried Cookie Test

Mice were initially habituated to a piece of cookie (McVities Hobnobs) introduced into their home cages on alternate days at least 5 days before the test commenced. Prior to the test, animals were food-restricted overnight (up to 16 h), although free access to water was maintained throughout this period. The test was conducted in Macrolon Type III cages (Tecniplast, Milan, Italy) measuring 36.9 × 16.5 × 13.2 cm illuminated by indirect lighting (~60 lux). The flooring of the cages was filled with corncob bedding to a depth of 2 cm and a piece of cookie was buried approximately 0.5 cm below the surface of the bedding. A transparent plastic lid with holes was positioned on top of the cage during testing to prevent the animal from climbing out. Animals were subjected to 3 trials with a maximum trial duration of 5 min per trial and an inter-trial interval of 1 min. Trial 1 was a habituation session without any cookie, whilst during trials 2 and 3, a piece of cookie was buried, with the position of the cookie switched and fully counterbalanced between trials to prevent any spatial bias. At the start of each trial, the animal was placed in the centre of the cage and allowed to explore freely, with their behaviour monitored by an overhead video camera and locomotor activity tracked by ANY-maze (version 5.1, Stoelting Co., Dublin, Ireland). The main parameter recorded was the latency for the animal to place both forepaws on the cookie piece during the trial duration; this was measured both manually by the experimenter using a stopwatch and automatically by the tracking software. A latency of 300 s was recorded if the subject failed to find the cookie within the allotted time. After completion of all trials, animals were returned to their home cages and given food immediately. The mean latency of trials 2 and 3 was calculated for each animal and used for analysis.

#### 2.3.6. Social Interaction

Social interaction and recognition of the mice was recorded using a three-chambered arena. The arena was constructed of white Perspex (63 cm × 42 cm × 22 cm) and consisted of two outer chambers (each 22 cm × 42 cm) and a central compartment (19 cm × 42 cm) separated by dividers with apertures allowing free movement between the chambers. A cylinder with metal bars was positioned in the centre of each of the outer chambers and used for the confinement of a stranger mouse during social interaction. The apparatus was illuminated by indirect overhead lighting during testing (~60 lux). Test sessions consisted of three phases: (i) habituation, (ii) sociability, and (iii) social memory/recognition. During habituation, the animal was placed in the central compartment and allowed to freely explore the arena for 10 min. In the sociability phase, an unfamiliar mouse (stranger 1 (S1)) of the same sex as the test mouse was placed inside one of the social interaction cylinders in either the left or right chamber, whilst the cylinder in the opposite chamber remained empty. The location of the stranger was randomly selected and counterbalanced (left or right) across groups. The test animal was again released in the central compartment and allowed to freely explore the arena over a 10 min test session. Measures taken included time spent within the immediate vicinity (4 cm interaction zone) of the two cylinders (S1 or empty). During the social memory test, a novel stranger was introduced into the second, previously empty cylinder and the exploration of the two cylinder zones (novel, unfamiliar stranger (S2) and now familiar stranger (S1)) were recorded for a further 10 min. The inter-trial interval between the different phases was 5 min and, between animals, the arena and cylinders were all cleaned and disinfected with 70% ethanol. The activity and exploratory behaviour of the mice were video-recorded and stored online (ANY-maze). Social interactions were quantified as time spent with S1 compared to the corresponding empty compartment (sociability), or with S2 compared to S1 (social memory). Distance travelled, time spent interacting (total), the time spent interacting with a stranger, and the discrimination index (ratio of time spent interacting with a stranger mouse to the total time spent interacting) were extracted/calculated and used for statistical analyses.

### 2.4. Tissue Collection

Brain tissue was harvested from all fifty-nine mice that underwent behavioural testing. Mice were euthanised via intraperitoneal injections of a sub-lethal dose of sodium pentobarbital (Dolethal (200 mg/mL), Covetrus, UK). Mice underwent intra-cardiac perfusion with heparinised phosphate-buffered saline (0.1 M PBS with 0.05% (*v*/*w*) heparin, pH 7.4) for 5 min. Skulls were then removed and the whole brain immediately dissected out. The right brain hemisphere was separated, fixed overnight at room temperature in 10% (*v*/*v*) neutral-buffered formalin, dehydrated, and embedded in paraffin. Sagittal sections were prepared at 5 µm using a rotary microtome (HM 325, Leica Biosystems, Sheffield, UK) and mounted onto glass slides (SuperFrost^TM^, Thermo Fisher Scientific, Lutterworth, UK). Sections were collected from regions at interaural 0.96 to 1.44 mm lateral of the midline [51], and three sections were collected on one slide for each mouse and antibody. The left-brain hemisphere was transferred immediately to liquid nitrogen after brain removal and kept at −80 °C until it was used for protein extraction/ELISA protein quantification.

### 2.5. Immunohistochemistry (IHC) and Quantification of Tau

Sagittal sections were stained in a sex-specific way using two immunohistochemistry staining boxes for males and two for female samples. Each box included a balanced number of L66 and L66 x mAPP^A673T^ mouse brains. Sections (three sections per mouse and antibody) were stained according to our standard protocol [36] using three different antibodies: the mouse monoclonal antibody mAb 7/51 targeting the epitope 350–368 within the microtubule domain of the tau protein (TauRx Therapeutics, Singapore, diluted 1:1000); the mouse monoclonal antibody HT7 targeting the epitope 159–163 within proline-rich domain of tau (Thermo Scientific, Loughborough UK, #MN1000, diluted 1:1000); and the mouse monoclonal antibody mAb 27/499 targeting the N-terminal epitope 14–26 (TauRx Therapeutics, diluted 1:200). All chemicals were purchased from Merck Millipore (Burlington, MA, USA). Images of cornu ammonis (CA1), the dentate gyrus (DG), the visual cortex (CTX), the prefrontal cortex (PFC), and the cerebellum (CB) were taken using a light microscope at a 100× magnification (Axio Imager M1, Carl Zeiss, Jena, Germany) and saved in TIFF file format. Microscopic images were analysed using ilastik (Version 1.4.0.post1, [52]) and Fiji (Version 2.14.0, [53]). The pixel classification tool in ilastik enabled training of the software based on a small subset of samples and then applied them to larger sets of images [52]. Models were trained to segment images into positively stained pixels and non-stained background tissue or artefacts. After applying models to all images, the percentage of positively stained area of the whole image was quantified using Fiji. This was repeated with each of the three antibodies for all fifty-nine mice.

### 2.6. Protein Extraction

All chemicals were purchased from Merck Millipore (Burlington, MA, USA) if not otherwise stated. The left hemibrains were pulverised in a liquid nitrogen prechilled stainless steel mortar and pestle (BioPulverizer, BioSpec, Bartlesville, OK, USA) and homogenised with a pestle and hammer. RIPA lysis buffer (Thermo Fisher Scientific, #89900) containing Pierce Protease and Phosphatase Inhibitor Mini Tablets (Thermo Fisher Scientific, # A32959) was added in a ratio of 5:1 (mL buffer to mg wet tissue) and the homogenate wasincubated for 30 min on ice with casual agitation. After centrifugation at 19,000× *g* for 2 h at 4 °C (Centrifuge 5427 R—Microcentrifuge, Eppendorf, Stevenage UK, using the FA-45-48-11 rotor), the supernatant (referred to as the RIPA-soluble supernatant fraction S1) was transferred into new reaction tubes. The residual pellet was homogenised in 5 volumes of tris-buffered saline (pH 7.6) containing 5 M guanidine hydrochloride (GuHCl) and incubated with mild agitation (11 rotations per minute, Multi Bio RS-24, Biosan, Riga, Latvia) for 16 h at room temperature. After centrifugation at 15,000× *g* for 30 min at room temperature, the resultant GuHCl supernatant fraction (referred to as S2) was transferred into new tubes. RIPA-soluble (S1) and RIPA-insoluble (S2) fractions were stored at −20 °C until use. Total protein concentrations of S1 and S2 fractions were determined by a bicinchoninic acid (BCA) protein assay (Pierce™ BCA Protein Assay Kit, Thermo Fisher Scientific, #23225) using bovine serum albumin (BSA) (0.125–2.000 mg/mL) as a reference standard.

### 2.7. Quantification of Tau, Aβ, and Synaptic Proteins Using ELISA

RIPA-soluble S1 was used to measure non-phosphorylated tau (non-pTau, ROBOSCREEN, Leipzig, Germany, #847-0108000102), Aβ40 (Invitrogen, Waltham, MA, USA, #KMB3481), Aβ42 (Invitrogen, #KMB3441), mouse synaptosomal-associated protein 25 kDa (SNAP25, MyBiosource, San Diego, CA, USA, #MBS451917), mouse syntaxin 1A (STX1A, MyBiosource #MBS452386), and mouse synaptophysin (SYP, MyBiosource #MBS453910). For non-pTau quantification, S1 samples were diluted 1:5000 in assay buffer. For Aβ40 and Aβ42, S1 samples were used undiluted. For synaptic protein ELISA, S1 samples were first diluted to a protein concentration of 4 µg/µL in RIPA and then further diluted in PBS, as recommended by the manufacturer, at a dilution of 1:2 for STX1A and 1:10 for SYP and SNAP25. Undiluted GuHCl S2 samples were used to assess aggregated tau (ROBOSCREEN, Leipzig, Germany, #847-0104000116). S2 samples were diluted 1:10 to measure Aβ40 and Aβ42 (same kits as above). ELISA assays were performed as per the manufacturer’s instructions. The levels for non-pTau, aggregated tau, SNAP25, SYP, and SNTX1A from all fifty-nine mice were analysed. For Aβ ELISA, some S1 and S2 samples had low sample volumes and could therefore not be included; where this is the case, sample sizes were amended in the figure legends.

### 2.8. Data Analysis

No a priori exclusion criteria were set. However, some IHC samples were excluded due to tissue damage during sectioning or lack of staining, possibly due to sample preparation error. Details are specified in the respective sections. All other data are included in the behavioural and cellular analysis and are presented here. Behavioural data were assessed for normality using the Shapiro–Wilk test and Gaussian distribution assumptions were met. Nest building scores were analysed using Aligned Rank Transform (ART) analysis of variance (ANOVA) with repeated measures and time, sex, and genotype as independent variables [54]. All other data were analysed using either 2-way ANOVA with genotype and time/trial, or sex and genotype as independent variables or 3-way ANOVA with genotype, sex and time as independent variables. GraphPad Prism software (version 10.2.3; GraphPad Software Inc., Boston, MA, USA) was used to generate graphs and conduct statistical analyses. IHC and ELISA data were analysed and graphs generated in R (Version 4.4.3, R Core Team, Vienna, Austria) using linear models and 2-way ANOVA. Where appropriate, post hoc tests were performed using Bonferroni corrections. For IHC staining, males and females were analysed separately and the effects of brain region, genotype, and their interaction on the percentage area stained were assessed. For each analysis, it was first determined whether data met assumptions for 2-way ANOVA (normality of residuals, heteroscedasticity) or whether data transformations were required. The data transformations used were square root, log or Box–Cox transformation and details are given in the respective figure legends. Data met necessary assumptions after transformation. A similar approach was taken for ELISA read-outs, where the effect of sex, genotype, and their interaction on protein levels were analysed. Due to the large number of samples, multiple ELISAs were performed, which in part were from different lots and performed on different days. This was accounted for by including a nuisance factor in the analysis for aggregated tau and synaptic proteins. All statistical outcomes are reported based on linear models of transformed data, but figures show untransformed raw data. For each genotype and sex, Pearson correlation matrices were generated from ELISA and IHC data and compared visually and statistically using the Jennrich test [55] to determine if the matrices were significantly different from each other. Extremely high values were removed from ELISA data to avoid skewing results and obtain correlations not driven by potential outliers. IHC data were averaged across brain regions for this analysis. All data are presented as Mean +/− Standard Deviation (S.D.) and alpha was set to *p* < 0.05.

## 3. Results

We have used L66 mice that overexpress the P301S aggregation-prone form of human tau and crossed them with mice carrying the A673T Icelandic mutation in the murine *APP* gene that is protective against Aβ aggregation in humans [42]. The experiments performed in this study were aimed at examining the effects of the Icelandic mutation on (i) tau and Aβ levels, (ii) synaptic protein abundance, and (iii) motor and neuropsychiatric behaviour in L66 and L66 x mAPP^A673T^ mice.

All mice were in good health with no obvious negative impact of tau overexpression or APP-A673T expression at the age of 6 months when they were investigated. The body weights did not differ significantly between genotypes (F_Genotype_(1, 55) = 1.00, *p* = 0.32) in male (L66: 39.5 ± 4.8 g vs. L66 x mAPP^A673T^: 37.8 ± 3.8) or female mice (L66: 32.3 ± 2.7 g vs. L66 x mAPP^A673T^: 32.1 ± 3.5). However, male mice were heavier than female mice (F_Sex_(1, 55) = 42.44, *p* < 0.0001), and this was the case in both genotypes (F_Interaction_ < 1).

### 3.1. Icelandic Mutation and Tau Pathology

**Figure 1:**
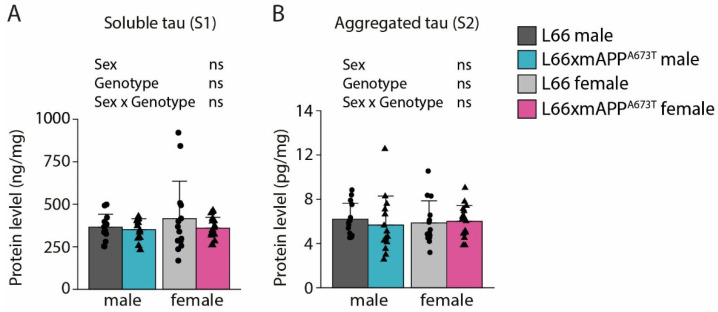
Soluble and aggregated tau ELISA. (**A**) Total non-phosphorylated tau was quantified in RIPA-soluble S1 fractions. (**B**) Aggregated tau was quantified in RIPA-insoluble (=GuHCl-soluble) S2 fractions. S1 and S2 fractions were prepared from whole-brain homogenates. Raw data for tau was normalised to protein levels and is shown as individual values, with group mean and S.D. Data were analysed using 2-way ANOVA with sex and genotype as independent variables and an additional nuisance factor (batch) for aggregated tau. Data were Box–Cox transformed for analysis. L66: males = 14, females = 14; L66 x mAPP^A673T^: males = 14, females = 17. Abbreviation: ns: not significant.

The levels of non-phosphorylated tau (non-pTau) and aggregated tau were measured in the RIPA-soluble and -insoluble fractions, respectively, of whole-brain homogenates using ELISA (Figure 1). Levels of non-pTau were similar in L66 and L66 x mAPP^A673T^ male mice and showed little variability (Figure 1A). While female L66 x mAPP^A673T^ mice also showed similar levels, female L66 had slightly higher levels and greater variability than the other three groups. Statistical analyses using two-way ANOVA did not return any significance (all F values < 1). The levels of aggregated tau in the RIPA-insoluble fraction were also similar across both genotypes and sexes, with average levels between 5.9 and 6.2 pg/mL for all groups (Figure 1B). Again, neither sex nor genotype or their interaction influenced aggregated tau levels (F values < 1).

**Figure 2:**
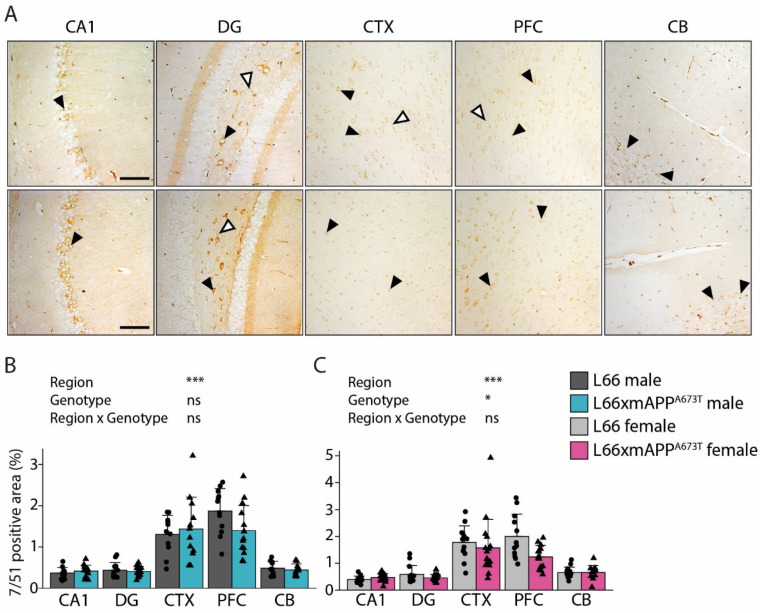
Tau immunohistochemistry using the microtubule domain mAb 7/51. (**A**) Representative tau immunohistochemistry images in brains of L66 (top) and L66 x mAPP^A673T^ (bottom) mice stained with the monoclonal anti-tau mAb 7/51. Images from CA1, DG, CTX, PFC, and the CB were taken using a light microscope at a 100× magnification. Black arrowheads = cytosolic staining; white arrowheads = axonal/dendritic staining; scale bars, 100 µm. Tau levels were quantified as stained area in percent using ilastik and Fiji and are shown for male (**B**) and female (**C**) mice as individual values, with group mean and S.D. Data were analysed using 2-way ANOVA with genotype and region as independent variables (* *p* < 0.05; *** *p* < 0.001). Data were log-transformed for analysis. Males: L66: *n* = 12 (DG *n* = 11, CB *n* = 10); L66 x mAPP^A673T^: *n* = 14 (CTX *n* = 12, CB *n* = 10). Females: L66: *n* = 14 (CTX *n* = 13, PFC *n* = 12); L66 x mAPP^A673T^: *n* = 16 (PFC *n* = 14, CB *n* = 12). Abbreviations: CA1: hippocampal CA1, CB: cerebellum, CTX: visual cortex, DG: dentate gyrus, ns: not significant, PFC: prefrontal cortex.

**Figure 3:**
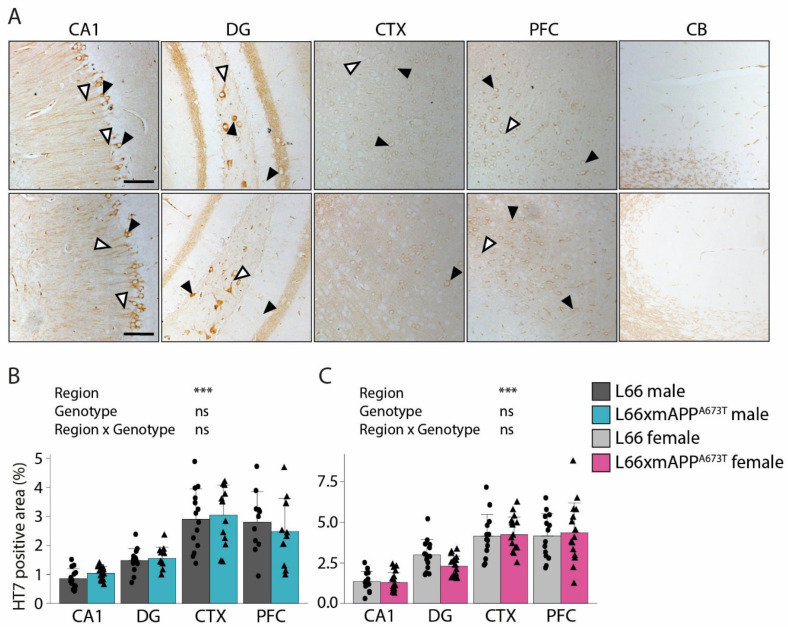
Tau immunohistochemistry using the proline-rich domain antibody HT7. (**A**) Representative tau immunohistochemistry images in brains of male and female L66 and L66 x mAPP^A673T^ mice stained with the monoclonal anti-tau antibody HT7. Images from CA1, DG, CTX, PFC, and the CB were taken using a light microscope at a 100× magnification. Black arrowheads = cytosolic staining; white arrowheads = axonal/dendritic staining; scale bars, 100 µm. Tau levels were quantified as stained area using ilastik and Fiji and are shown for male (**B**) and female (**C**) mice as individual values, with group mean and S.D. Data were analysed using 2-way ANOVA with genotype and region as independent variables (*** *p* < 0.001). Data were log-transformed for analysis. Males: L66: *n* = 14 (PFC *n* = 11); L66 x mAPP^A673T^: *n* = 14 (DG *n* = 11, CTX *n* = 13, PFC *n* = 12). Females: L66: *n* = 14; L66 x mAPP^A673T^ *n* = 17 (PFC, CTX *n* = 16). Abbreviations: CA1: hippocampal CA1, CB: cerebellum, CTX: visual cortex, DG: dentate gyrus, ns: not significant, PFC: prefrontal cortex.

**Figure 4:**
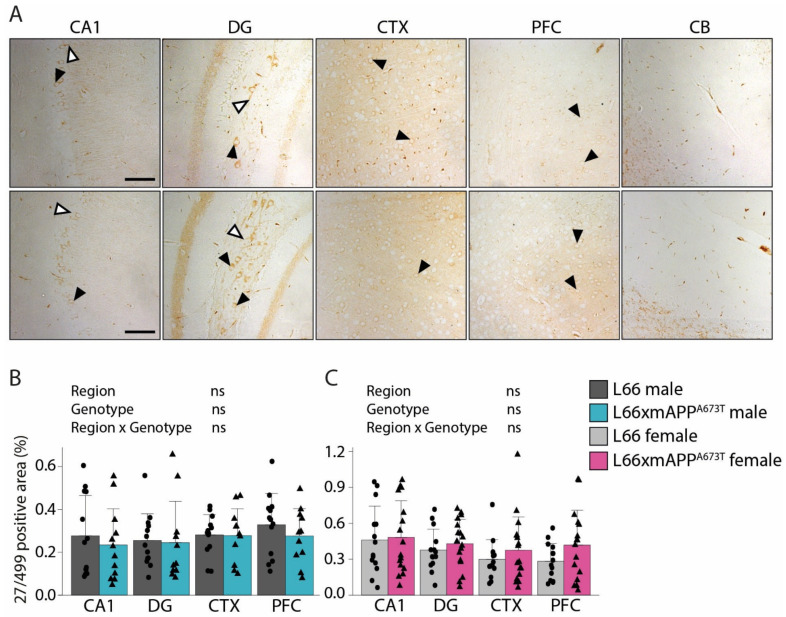
Tau immunohistochemistry using the N-terminal domain antibody 27/499. (**A**) Representative tau immunohistochemistry images in brains of male and female L66 and L66 x mAPP^A673T^ mice stained with the monoclonal anti-tau antibody 27/499. Images from CA1, DG, CTX, PFC, and the CB were taken using a light microscope at a 100×. Black arrowheads = cytosolic staining; white arrowheads = axonal/dendritic staining; scale bars, 100 µm. Tau levels were quantified as stained area using ilastik and Fiji and are presented for male (**B**) and female (**C**) mice as individual values, with group mean, and S.D. Data were analysed using 2-way ANOVA with genotype and region as independent variables. No data transformation was required. Males: L66: *n* = 13; L66 x mAPP^A673T^: *n* = 12, (DG, CTX, PFC *n* = 11). Females: L66: *n* = 14; L66 x mAPP^A673T^: *n* = 17 (CA1, PFC *n* = 16). Abbreviations: CA1: hippocampal CA1, CB: cerebellum, CTX: visual cortex, DG: dentate gyrus, ns: not significant, PFC: prefrontal cortex.

To analyse tau on a brain-region level, immunohistochemistry was performed using antibodies that target different epitopes of tau, and the positive signal was quantified as percentage of stained area in five regions of interest: CA1, DG, CTX, PFC, and CB (Figure 2, Figure 3 and Figure 4).

The phosphorylation-independent mAb 7/51 binds to residues 350–368 within the microtubule binding domain and the core fragment of tau in the paired helical filament (PHF) typical for AD brain tissue. Positive staining with 7/51 showed a similar pattern in both L66 and L66 x mAPP^A673T^ crosses, with prominent staining in the soma of pyramidal cells in CA1, DG hilus and CTX as well as in large Purkinje cells of the CB (Figure 2A, black arrowheads). Less prominent dendritic staining was also observed in hippocampal and visual and prefrontal cortical areas (Figure 2A, white arrowheads). In male mice (Figure 2B), there was regional variation in mAb 7/51-positivity, with the greatest levels being observed in cortical areas (F_Brain region_(4, 111) = 72.22, *p* < 0.001). Reactivity was similar in L66 and L66 x mAPP^A673T^ mice (F_Genotype_ < 1), with no obvious interaction between genotype and region as factors (F_Interaction_(4, 111) = 1.14, *p* = 0.34). Nonetheless, a trend towards decreased 7/51-reactivity was observed in PFC (25%). In female mice (Figure 2C), staining intensity was again highest in cortical regions (F_Brain region_(4, 131) = 61.15, *p* < 0.001). Importantly, female L66 x mAPP^A673T^ crosses exhibited less 7/51-reactive tau than L66 females overall (F_Genotype_(1, 131) = 4.47, *p* = 0.036), and this difference was most notable in PFC (38%).

HT7 is a further phosphorylation-independent anti-tau antibody which recognises an epitope in the proline-rich region of the tau protein, a region involved in the interaction of tau with actin and other cytoskeletal proteins. Intraneuronal staining in the CA1, hilus, and granular cell layer of the DG, as well as the visual and prefrontal cortex, was observed; staining patterns were similar in L66 and L66 x mAPP^A673T^ crosses (Figure 3A, black arrowheads). Processes were also frequently labelled in CA1, but less prominently in DG and cortical regions (Figure 3A, white arrowheads). The CB was devoid of any labelling. The staining intensity with HT7 was greatest in CTX and PFC, both in male (F_Brain region_(3, 95) = 52.33 *p* < 0.001, Figure 3B) and female mice (F_Brain region_(3, 114) = 66.99, *p* < 0.001, Figure 3C), confirming the staining pattern already seen with mAb 7/51 (see above). Furthermore, no differences were observed between L66 and L66 x mAPP^A673T^ crosses, either in male or female cohorts (all F values < 1).

The N-terminal, phosphorylation-independent anti-tau antibody 27/499 binds the epitope of tau known to interact with cytoplasmic components/proteins. The staining pattern with this antibody resembled the pattern revealed by the two antibodies described above, although the intensity was slightly weaker overall and completely absent in CB (Figure 4A). Additionally, the staining did not differ across brain regions, either in males (Figure 4B) or females (Figure 4C). Like the HT7 labelling reported above, no differences for 27/499-reactive tau were observed between L66 and L66 x mAPP^A673T^crosses in male or female cohorts.

### 3.2. Icelandic Mutation, Aβ, and Synaptic Proteins

**Figure 5:**
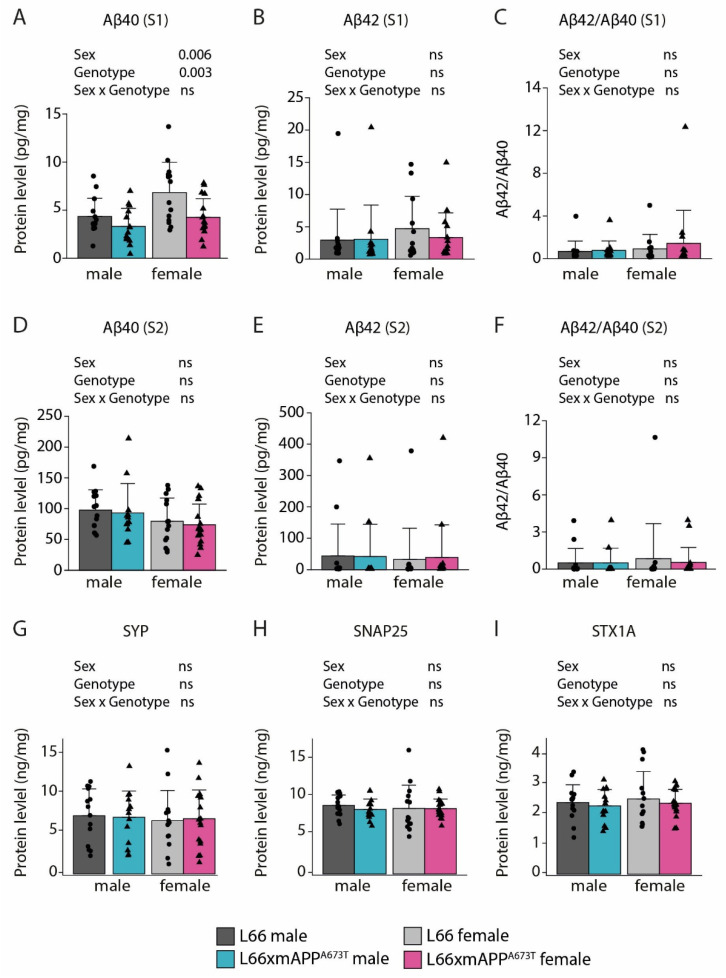
Quantification of Aβ and synaptic proteins in L66 and L66 x mAPP^A673T^ male and female mice. Mouse Aβ40, Aβ42 and their Aβ42/Aβ40 ratio were quantified in RIPA-soluble (**A**–**C**) and -insoluble fractions (**D**–**F**) prepared from whole-brain homogenates. (**G**) SYP, (**H**) SNAP25, (**I**) and STX1A were quantified in RIPA-soluble S1 fractions. Raw data for Aβ and synaptic proteins were normalised to protein levels and are shown as individual values, with group mean and S.D. Data were analysed using 2-way ANOVA, with sex and genotype as independent variables. Aβ data were square root- (**A**–**D**), or Box–Cox (**E**,**F**)-transformed for analysis. Synaptic protein data (**G**–**I**) did not require transformation. Where samples could not be included due to low sample volume (Aβ quantification), this is indicated in sample sizes in the brackets below. L66: males = 14 (S1 Aβ42:13), females = 14; L66 x mAPP^A673T^: males = 14 (S2 Aβ40:12, S2 Aβ42:13), females = 17. Abbreviations: ns: not significant, SNAP25: synaptosomal-associated protein 25kDa, STX1A: syntaxin 1A, SYP: synaptophysin.

To assess the effects of the protective Icelandic mutation on Aβ production in L66 and L66 x mAPP^A673T^ crosses, Aβ40 and Aβ42 were analysed in S1 and S2 fractions. Aβ40 in S1 fractions was lower in L66 x mAPP^A673T^ compared to L66 (F_Genotype_(1, 55): 9.51, *p* = 0.003; Figure 5A), while Aβ42 and Aβ42/Aβ40 ratios in the same fraction were similar in both sexes and genotypes (Figure 5B,C). Furthermore, no differences were observed between genotypes for the RIPA-insoluble Aβ40, Aβ42 and Aβ42/Aβ40 ratio in S2 fractions (Figure 5D–F). Interestingly, Aβ40 in S1 was greater in female mice compared to male mice in both genotypes (F_Sex_(1, 55) = 8.32, *p* = 0.006; Figure 5A). Having established a small, yet significant, reduction in tau and soluble Aβ40 in L66 x mAPP^A673T^, we further explored whether this reduction may lead to changes in synaptic protein expression using a set of three presynaptic proteins, SYP, SNAP25, and STX1A, that were quantified by ELISA. The levels of SYP showed a high degree of variability but were overall similar across genotypes and sexes (F values < 1; Figure 5G). SNAP25 had slightly higher abundance than SYP (SNAP25: 8.85 ± 1.89 ng/mg vs. SYP: 7.18 ± 3.52 ng/mg) and showed lower variability (Figure 5H). Again, there were no significant differences in SNAP25 levels between L66 and L66 x mAPP^A673T^, and the levels were also comparable between male and female cohorts (independent of genotype, all F values < 1). The least abundant protein was STX1A, with an average level of 2.34 ± 0.62 ng/mg (Figure 5I) and, as with the other two synaptic proteins, the levels of STX1A were comparable between all cohorts (F values < 1).

**Figure 6:**
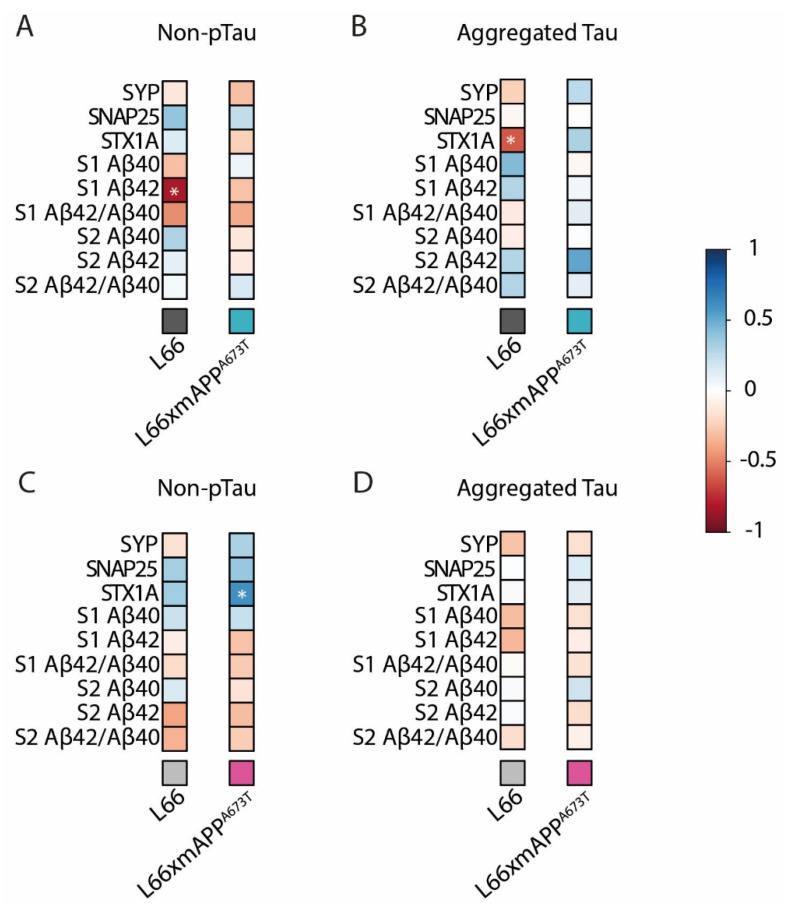
Correlation matrices between tau, Aβ and synaptic proteins. Pearson correlation matrices between non-pTau and aggregated tau with the synaptic proteins SYP, SNAP25, STX1A, as well as Aβ40, A β42, and their ratio in S1 and S2 are displayed for L66 male (**A**), L66 x mA673T male (**B**), L66 female (**C**), and L66 x mA673T female (**D**) with blue for positive correlations, red for negative correlations, and white where no correlation was seen (* *p* < 0.05). SYP, SNAP25, STX1A, non-pTau, aggregated tau, Aβ40, and A β42 were quantified in S1 and S2 brain homogenate fractions using ELISA. HT7-tau, 27/499-tau, and 7/51-tau were quantified using immunohistochemistry (averaged across brain regions). Data were analysed using the Jennrich test to detect differences between matrices. Abbreviations: SNAP25: synaptosomal-associated protein 25kDa, STX1A: syntaxin 1A, SYP: synaptophysin.

Pearson correlation matrices were generated to further investigate any differences between genotypes in terms of correlations between tau and Aβ pathology and synaptic protein markers. In male mice, there was a difference between L66 and L66 x mAPP^A673T^ correlation matrices both visually and statistically (Figure S1A,B, *p* < 0.001; see the Supporting Information). Similarly, correlation matrices of female L66 and L66 x mAPP^A673T^ were also significantly different (Figure S1C,D, *p* < 0.001; see the Supporting Information). A direct comparison in terms of amyloid and synaptic markers with aggregated and non-pTau levels can be obtained from correlation matrices comparing males and female mice of the different genotypes (Figure 6A–D). While in L66 males, non-pTau and Aβ42 levels in S1 showed negative correlations (Figure 6A; see the asterisks), this correlation was lost in L66 x mAPP^A673T^ males. Meanwhile, aggregated tau levels did not correlate with any of the Aβ measures in either genotype (Figure 6B). Further, a strong negative correlation between aggregated tau and STX1A was seen in L66 but not L66 x mAPP^A673T^ (Figure 6B; see the asterisks). In females, no correlations were seen between Aβ and non-pTau (Figure 6C), or Aβ and aggregated tau (Figure 6D).

### 3.3. Icelandic Mutation and Behaviour

**Figure 7:**
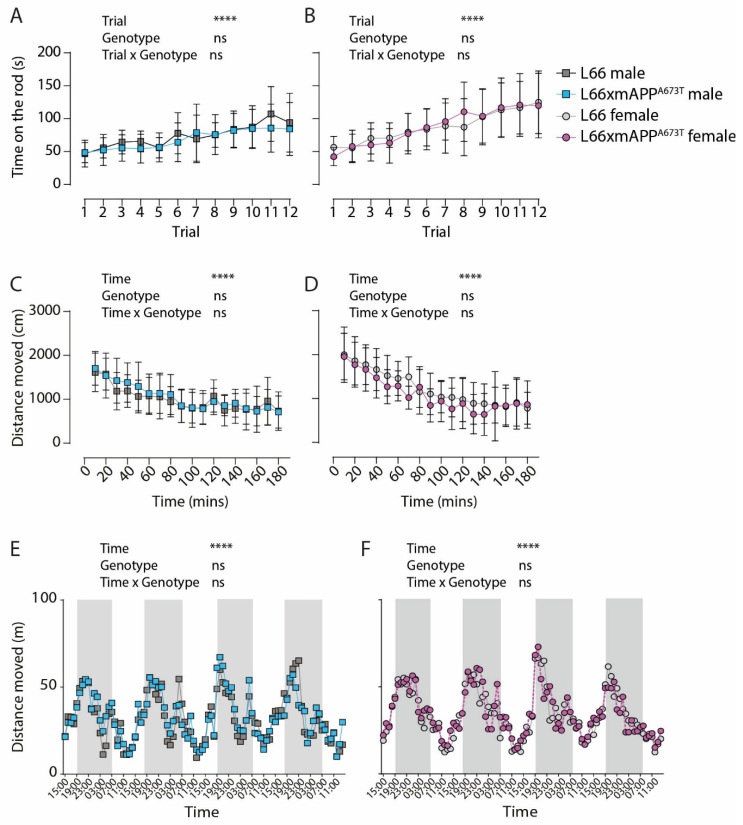
Behaviour on the rotarod and in the PhenoTyper of L66 and L66 x mAPP^A673T^ mice. Motor function was assessed using a four-lane rotarod system. Each mouse was given a total of 12 trials (T1-T12) over three days and the time spent on the rod was plotted for male (**A**) and female (**B**) mice. Activity in the PhenoTyper during the first 180 min (habituation) was assessed as distance moved for male (**C**) and female (**D**) mice. Additionally, the circadian activity (distance moved) over weekdays was quantified in hourly bins in the PhenoTyper for male (**E**) and female (**F**) mice and the active (dark) phase from 7 pm to 7 am is highlighted in grey. No differences between genotypes were observed between genotypes in any metric. Data are shown as either group mean and S.D. (**A**–**D**), or as group mean only for clarity (**E**,**F**). Data were analysed using 2-way ANOVA with genotype and trial (**A**,**B**) and genotype and time (**C**–**F**) as independent variables (**** *p* < 0.0001). Abbreviation: ns: not significant.

**Figure 8:**
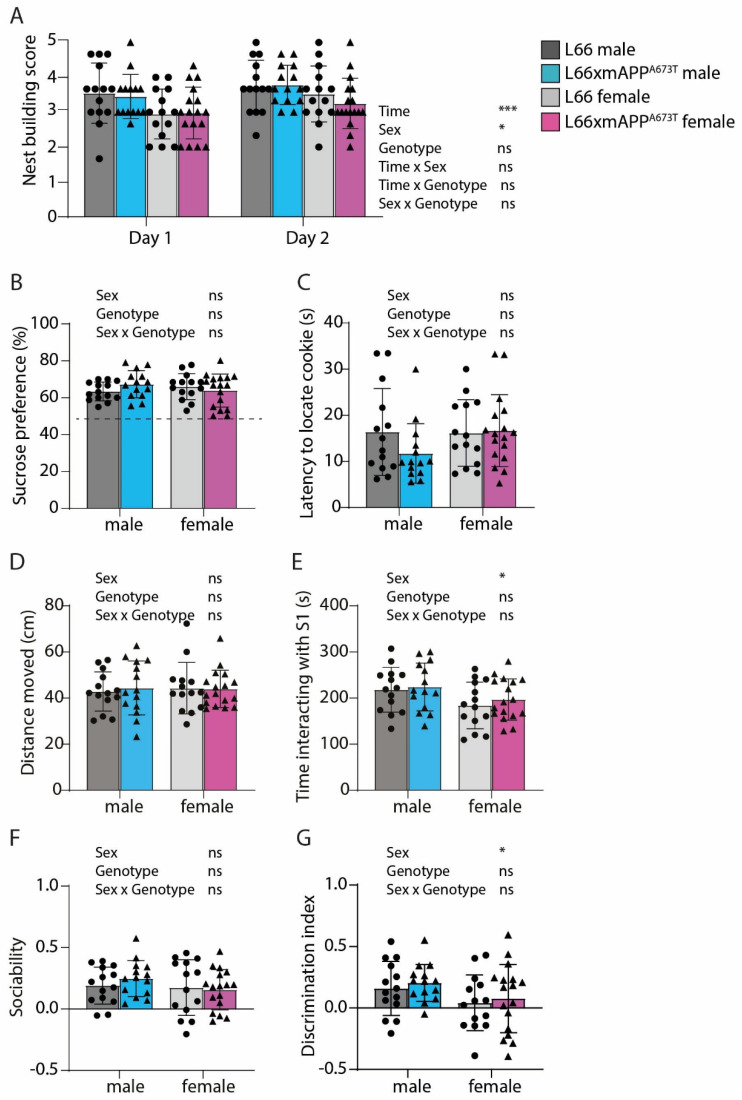
Behaviour during nestbuilding, sucrose preference, buried cookie, and social interaction tests of L66 and L66 x mAPP^A673T^ mice. Nestbuilding ability was assessed after a period of 16 h (Day 1) and 48 h (Day 2) following the introduction of the nestlet into home cages (**A**). Anhedonic-like behaviour and olfaction were assessed using the sucrose preference test (**B**) and the buried cookie test (**C**), respectively. Lastly, for the social interaction test, distance moved was determined during habituation (**D**), time spent interacting with the S1 stranger during the sociability phase (**E**), and time spent with novel stranger S2 (**F**), and the discrimination index (**G**) were analysed for the social recognition phase. No genotype-related differences were recorded. Data are shown as individual values, with group mean and S.D., and were analysed using repeated measures ART ANOVA with time, sex, and genotype as independent variables ((**A**), * *p* < 0.05; *** *p* < 0.001), or 2-way analysis of variance (ANOVA) with sex and genotype as independent variables ((**B**–**G**), * *p* < 0.05). Dotted line in B indicates 50% level of chance. Abbreviation: ns: not significant.

We also examined whether any pathological differences between and L66 and L66 x mAPP^A673T^ mice may translate to functional consequences in terms of behaviour and thus tested these mice using six different paradigms assessing motor (Figure 7) and neuropsychiatric symptoms, including social memory (Figure 8), known to be associated with dementia. Tests were performed in the sequence described here.

Motor competence was examined using the rotarod. Motor performance, expressed as the time spent on the rotating rod, was similar for L66 and L66 x mAPP^A673T^ male (F values < 1; Figure 7A) and female cohorts (F values < 1; Figure 7B). Similarly, motor learning, identified as increased time on the rod between trials and sessions, occurred in all cohorts (males F_Trial_(4.405, 114.5) = 11.69; females F_Trial_(5.277, 153.0) = 25.18; *p* values < 0.0001).

Home cage activity offers the possibility for long-term monitoring and the ability to assess and track behaviour in a relatively stress-free environment, and this was done in the current study using the PhenoTyper home cage observation system [56]. The first three hours in the PhenoTyper are considered as habituation to a novel environment and the distance moved during this time is plotted separately as a function of time. Male mice of both genotypes habituated similarly to the new environment, as shown by the decreased distance covered over time (F_Time_(6.046, 151.1) = 17.30, *p* < 0.0001; Figure 7C), and there were no significant differences between L66 and L66 x mAPP^A673T^ males (F_Genotype_(1, 25) = 0.32, *p* = 0.58). Female mice also showed similar habituation over time (F_Time_(5.907, 159.5) = 30.07, *p* < 0.0001; Figure 7D), and, again, no differences between genotypes were observed (F_Genotype_(1, 27) = 1.31, *p* = 0.26). Long-term monitoring of the average distance travelled in a 24-h period over the 4 days of home cage observation also did not yield significant differences between L66 and L66 x mAPP^A673T^ males (F_Genotype_(1, 2304) = 1.16, *p* = 0.28; Figure 7E) or females (F_Genotype_(1, 2588) = 0.50, *p* = 0.48; Figure 7F). All cohorts showed typical circadian rhythms with heightened peak ambulatory activity during the dark phase (7 p.m. until 7 a.m. highlighted in grey) and are more sleep-prone during the light phase (males F_Time_(95, 2304) = 9.48; females F_Time_(95, 2588) = 12.62; *p* values < 0.0001).

The neuropsychiatric assessment included nestbuilding, sucrose preference, buried cookie, and social interaction testing (Figure 8).

Apathy-like behaviour was measured using the nestbuilding test [38]. Readings were taken at 24 and 48 h after the introduction of nestlets using a five-point scale. After 24 h, all mice achieved a score between 2 and 5, with a score of 1 indicating absence of any nestlet shredding and a score of 5 indicating complete shredding and perfect nest construction. No significant differences were observed between genotypes (Figure 8A, F_Genotype_ < 1), but male mice achieved significantly higher scores than female mice (F_Sex_(1, 55) = 6.06, *p* = 0.017). Additionally, all cohorts improved their nestbuilding abilities at 48 compared to 24 h (F_Time_(1, 55) = 13.48, *p* = 0.0005). A lack of differences in anhedonia between L66 and L66 x mAPP^A673T^ mice was further confirmed using the sucrose preference test [38]. Data on fluid consumption revealed that all groups preferred sucrose over water, with one-sample *t*-tests confirming that the preference for L66 and L66 x mAPP^A673T^ crosses was significantly above the 50% level of chance (Figure 8B, all *p* values < 0.0001). No overall genotype or sex differences were observed for either sucrose preference (F values < 1) or total fluid intake. The buried cookie test was used to assess olfactory ability and motivation (Figure 8C). As no overall differences were observed between the readouts from manually timed assessments and the automated tracking software, they both were deemed to be equally reliable. Therefore, the automated testing was used for the purpose of this analysis. No genotype or sex differences were observed in the mean latency to locate the cookie during the two buried cookie trials (trials 2 and 3), with all groups locating the cookie within an average of 11–17 s (F values < 1). Further analysis confirmed sex differences, with the L66 x mAPP^A673T^ males presenting with heightened activity compared to the females (*p* = 0.0017). The results from social interaction and recognition testing are summarised in Figure 8D–G. During the habituation phase, all mice presented with similar levels of locomotor activity, with no overall differences observed between genotypes or sexes (F values < 1, see Figure 8D). Similarly, all groups spent a considerable amount of time adjacent to the cylinder containing the stranger mouse S1 during the sociability phase (Figure 8E). This amount of time was considerably higher than the time spent with the empty cylinder (main effect of zone: (F(1, 55) = 72.62, *p* < 0.0001) and this is reflected in the discrimination index for the sociability phase (Figure 8F). While there was no main effect of sex, genotype, or interaction (F’s < 1.3), all groups displayed a significant bias the interaction zone of S1 (one-sample *t*-test, all *p*s < 0.013).

For social memory, the time bias for stranger S2 was above chance only for the males, but not the females (ts > 2.5, *p*s < 0.02 for males; ts < 1.2, ns for females). Globally, there were no differences observed between L66 and L66 x mAPP^A673T^ animals for the discrimination index (F < 1) and a main effect of sex occurred (F(1, 55) = 4.2, *p* = 0.04) only occurred with regard to the time spent interacting with the S1 mouse (Figure 8E). During the social recognition test, all genotypes/sexes displayed a preference for the novel stranger mouse S2, spending an increased amount of time exploring S2 compared to the familiar S1 mouse (F(1, 55) = 17.28, *p* < 0.0001), with no genotype differences for time spent with the novel S2 mouse (Figure 8F) or the discrimination index (Figure 8G). Sex differences were apparent during the test, with both L66 and L66 x mAPP^A673T^ female mice displaying reduced overall exploration compared to their male counterparts (*p* values < 0.0001). Female mice of both genotypes also spent less time interacting with the S1 mouse relative to the male mice (F(1, 55) = 5.78; *p* = 0.02; Figure 8E). Further sex differences were also present, where female mice had a lower discrimination index than male mice (F(1, 55) = 4.244; *p* = 0.0441; Figure 8G).

## 4. Discussion

The APP^A673T^ Icelandic mutation has been shown to reduce the risk of AD through reducing Aβ production [42,43]. However, its effect on tau pathology is not well studied and has never been investigated in tau transgenic mice. In this study, we have therefore examined whether the addition of the A673T mutation to the murine *APP* gene can directly reduce tau pathology and thereby rescue behavioural deficits previously reported for L66 tau transgenic mice [37,38].

### 4.1. mA673T Aβ Has a Marginal Impact on Tau Pathology and Soluble Aβ40 Levels in L66 Mice In Vivo

L66 mice overexpress the aggregation-prone mutant form P301S of the longest human tau isoform. The mice show tau accumulation in cells in various brain regions and the aggregated state for some of these tau species has been verified by binding to Bielschowsky silver and primulin [37]. Furthermore, synaptic accumulation of tau in L66 mice has been confirmed by immunohistochemical co-localisation with synaptic markers and by co-purification with synaptic proteins in synaptosomal and synaptic vesicle preparations [36,57]. The reactivity of the various tau species in L66 has been extensively investigated histopathologically using a large set of monoclonal antibodies. These include mAb 7/51 targeting an epitope within the microtubule domain, HT7 targeting an epitope within proline-rich domain of tau, and mAb 27/499 targeting its N-terminal domain [36]. These three antibodies have been applied in the current study to enable a thorough examination of the abundance of distinct tau species in different regions of the L66 brain [36,37]. However, here, we report minimal differences in tau species in L66 x mAPP^A673T^ crosses compared with L66 tau mice. We found a significant reduction in 7/51-reactive tau in female L66 x mAPP^A673T^ crosses compared to L66. The reduction was most obvious in PFC in the range of 25 and 38% (in males and females, respectively), but no further differences between genotypes and the other antibodies emerged. This is surprising given that the three antibodies, 7/51, HT7 and 27/499, span a number of domains across the tau molecule and L66 mice, showing intense immuno-positive labelling that is absent from non-transgenic wild-type control mice [36]. We take this as a lack of efficacy of the mAPP^A673T^ mutation to mechanistically counter the overexpression of tau in 6-month-old L66 mice. This was confirmed for soluble and insoluble fractions in which no overt differences in tau levels were observed between genotypes. The ELISAs used to measure such changes were highly specific for L66 tau relative to non-transgenic wild-type mice (unpublished data).

Previous work has suggested that the APP^A673T^ Icelandic mutation can protect against Aβ pathology in Aβ-transgenic mice and Aβ-knock-in rats [47,58]. In our model, in which the mutation was introduced into the murine *APP* gene and the resultant mice crossed with L66, soluble mouse Aβ40 was reduced by 23 to 38% (in males and females, respectively) and this reduction in Aβ40 is in line with the reduction seen for mAb 7/51-reactive tau in PFC. Intriguingly, a recent study used APPswe/PS1dE9 transgenic mice and inoculated them with either recombinant non-mutant human Aβ or human Aβ containing the A673T mutation. No changes in transgenic Aβ levels were observed, but there was a decrease in endogenous, mouse phospho-tau pathology in the A673T-Aβ-treated group, and this remains unexplained [48]. In our study, we found that mAPP^A673T^ had only a small impact on transgenic, human tau in L66 mice. Here, we did not measure phospho-tau in L66 brains because the aggregation of non-phosphorylated tau is correlated with the onset of cognitive impairment in mice [37], and the phosphorylation of tau inhibits tau–tau binding and is preceded by aggregation of non-phosphorylated tau [59,60]. These latter data strongly suggest a prominent role of phosphorylation-independent processing of tau in both its aggregation and the associated decline in cognition. A recent exploratory study in six non-AD patients (unconfirmed idiopathic normal pressure hydrocephalus cases) comparing the CSF of three APP^A673T^ carriers to three age- (and sex-) matched control subjects reported that disease-relevant soluble APP-β and Aβ42 levels were significantly reduced in CSF of APP^A673T^ carriers. Yet, soluble APP-α, Aβ40, total tau and phosphorylated tau (p-tau 181) were not altered [61], which also brings into question the efficacy of the ‘protective mutation’ in terms of tau expression. While this small study cohort needs verification, the work seems to suggest that detection of protective A673T amyloid levels may differ between compartments and is specific to CSF. We did not investigate CSF/plasma tau here, but reasoned that detection of disease-relevant markers directly at the source, i.e., in neurones and within the brain, may be a more appropriate means for differentiation of phenotypes. Moreover, recent work in L66 has shown a highly significant correlation between insoluble brain tau and p-tau 217 plasma tau [62]. Consequently, CSF tau in L66 would reflect brain tau and is unlikely to be reduced following the introduction of the mAPP^A673T^ mutation in our L66 tau transgenic line. Interestingly, the mA673T mutation also altered correlations between the Aβ and both non-pTau and aggregated tau. This would suggest that mAPP^A673T^ has unique effects on tau pathology that differ from wildtype murine Aβ, which is interesting, considering that both proteins can act in complex synergy [63]. Since a reduction in endogenous murine *APP* exacerbated tau pathology in an APP-knockout and tau-overexpressing mouse model (Tg30) [64], it is not inconceivable that small reductions in Aβ40/42, as seen in our L66 x mAPPA673T crosses, do not reduce tau production and are unable to rescue either tau pathology or the behavioural responses of L66 mice.

### 4.2. mA673T Aβ Does Not Modify Synaptic Protein Expression in L66 In Vivo

Synaptic dysfunction that contributes to memory impairment and synapse loss, confirmed by postmortem analyses of AD brain tissue, is the strongest correlate for cognitive decline [65,66,67]. The presynaptic proteins SYP and SNAP25 were chosen as established markers for synapse loss in AD, as well as in tau- and Aβ-based mouse models [65,66,68]. Our previous work established that tau aggregation induces alterations in synaptic proteins and synaptic transmission in L66 mice [39]. This made it reasonable to confirm whether the protective mutation in the *APP* gene could correct the abnormal levels of synaptic proteins. Six-month-old L66 mice are particularly sensitive to reductions in structural and release-competent presynaptic proteins such as SNAP25 and STX1A, and reduction in tau using a small-molecule aggregation inhibitor resulted in partial recovery of normal synaptic protein expression [39,40]. In the current study, mAPP^A673T^ did not seem to modulate the expression of the three investigated presynaptic proteins SYP, SNAP25 and STX1A. This is likely due to a lack of substantial modulation of tau and/or Aβ in the L66 x mAPP^A673T^ crosses. However, there was a strong negative correlation between levels of aggregated tau and STX1A in L66 male mice that was absent in L66 x mAPP^A673T^. In female L66 x APP^A673T^, there was an enhanced positive correlation between STX1A and non-pTau compared to L66. The lack of negative association between STX1A and aggregated tau could suggest a small protective effect of L66 x mAPP^A673T^, making synapses less sensitive to synaptic protein loss in response to tau aggregation. No such association was seen for either genotype in females, suggesting there may also be a sex-specific component to this.

### 4.3. mA673T Aβ Does Not Modulate Motor and Neuropsychiatric Phenotypes in L66 Tau Transgenic Mice

The APP^A673T^ mutation was also associated with increased cognitive performance in carriers without AD compared to noncarriers based on the Cognitive Performance Scale, suggesting a possible protective effect of the Icelandic mutation that may be unrelated to AD and/or Aβ/tau pathologies [43]. This notion was based on a case study of a cognitively normal centenarian with vascular amyloid pathology and Braak stage 3 tau pathology [69]. We therefore reasoned that our L66 x mAPP^A673T^ crosses could benefit from this mutation independent of a potential normalisation of dementia-related pathology and thus performed a behavioural phenotyping study despite the lack of any substantial tau and/or Aβ reduction. Using six different behavioural tests addressing motor function, olfaction, depression and apathy-like behaviour, as well as motivation, exploration and sociability/social memory, we failed to find any overt differences between L66 and L66 x mAPP^A673T^ crosses. While not in line with our hypothesis, it needs to be considered that only very few studies have investigated the effect of the Icelandic mutation on behaviour in mice and the results are inconsistent. For example, inoculation of A673T Aβ into Aβ-transgenic mice rescued spatial memory deficits in the Morris water maze via a reduction in tau but not Aβ pathology [48]. A related study investigated the APP^A673V^ mutation, which has previously been described to reduce Aβ if expressed in a heterozygous background [70,71]. After induction of a traumatic brain injury in the 3xTg-AD model, mice were inoculated with vehicle or APP^A673V^. Improved locomotor activity in the open field and cognitive behaviour in the Y-maze were amongst the beneficial effects induced by the protective Aβ peptide [72]. Discrepancies in behavioural outcomes are most likely related to the use of distinct AD models (APPswe/PS1dE9 [48], 3xTg-AD [72] or L66 in the current study), but also to the different approaches of APP^A673T^ delivery (inoculation vs. genetic expression). One further possible explanation is the use of mouse versus human APP^A673T^. Aβ from mouse and human differs by three amino acids, two of which are located close to the beta-secretase 1 (BACE1) cleavage site that initiates the production of Aβ from APP [34]. However, it has been shown that overexpression of murine Aβ can produce amyloid deposits [34], and knock-in of human BACE1 leads to amyloid deposits purely based on murine Aβ [73]. These data confirm the propensity of murine Aβ for aggregation but, so far, no systematic and confirmatory comparison between these approaches is available. Moreover, earlier work revealed that the accumulation of tau in L66 leads to sensorimotor/motor deficiencies and neuropsychiatric symptoms, e.g., using rotarod, sucrose preference, and nestbuilding paradigms at 6 months of age, but not a cognitive phenotype [37,38]. It is therefore conceivable that benefits exerted by APP^A673T^ are specific to memory functions not examined here, yet which require further study.

## 5. Conclusions

In summary, we here show that the APP^A673T^ Icelandic mutation does not modulate motor and neuropsychiatric behaviours in L66 tau transgenic mice, possibly due to the small modification of tau pathology in 6-month-old L66 x mAPP^A673T^ mice.

## Figures and Tables

**Table 1:** Study groups and cohort sizes. L66: heterozygous line 66 mice; L66 x mAPP^A673T^: crosses carrying both the L66 tau-transgene and the A673T mutation in the murine *APP* gene. N: number of mice.

	N Male (Cohort 1 and Cohort 2)	N Female (Cohort 1 and Cohort 2)
L66	14 (7 and 7)	14 (7 and 7)
L66 x mA673T	14 (7 and 7)	17 (9 and 8)
N—Total	∑ 59	

## Data Availability

The raw data supporting the conclusions of this article will be made available by the authors on request.

## Supplementary Materials

The following supporting information can be downloaded at: https://www.mdpi.com/article/10.3390/biom16010028/s1, Figure S1: Correlation matrices between tau, Aβ and synaptic proteins of L66 and L66 x mAPP^A673T^ mice.

## Abbreviations

AD: Alzheimer’s disease; APP: amyloid precursor protein; ART: Aligned Rank Transform; Aβ: amyloid β-protein; BCA: bicinchoninic acid; CA1: cornu ammonis; CB: cerebellum; CTX: visual cortex; DG: dentate gyrus; IHC: immunohistochemistry; L66: line 66 tau transgenic mice; mAb: monoclonal antibody; non-pTau: non-phosphorylated tau; PFC: prefrontal cortex; S.D.: standard deviation; SNAP25: synaptosome-associated protein 25kDa; STX1A: syntaxin 1A; SYP: synaptophysin.
