# Supplementary material for: Effect of the Icelandic Mutation APPA673T in the Murine APP Gene on Phenotype of Line 66 Tau Mice
Source: Biomolecules. 2025 Dec 24;16(1):28. doi: 10.3390/biom16010028 (PMC12838599; doi:10.3390/biom16010028)
Supplement: Supplementary file 1 [file biomolecules-16-00028-s001.zip › biomolecules-4031468-supplementary.pdf]

# SUPPORTING INFORMATION

# Effect of the Icelandic Mutation APP$^{A673T}$ in the Murine APP Gene on Phenotype of Line 66 Tau Mice

**Anne Anschuetz [1], Lianne Robinson [1], Miguel Mondesir [1], Valeria Melis [1], Bettina Platt [1], Charles R. Harrington [1,2], Gernot Riedel [1,*,†] and Karima Schwab [1,†]**

[1] School of Medicine, Medical Sciences and Nutrition, University of Aberdeen, Foresterhill, Aberdeen AB25 2ZD, UK

[2] TauRx Therapeutics Ltd., 395 King Street, Aberdeen AB24 5RP, UK

* Correspondence: g.riedel@abdn.ac.uk

† These authors contributed equally to this work.

**Key words:** Tau, amyloid-beta, Icelandic mutation, dementia, synaptic proteins, behaviour

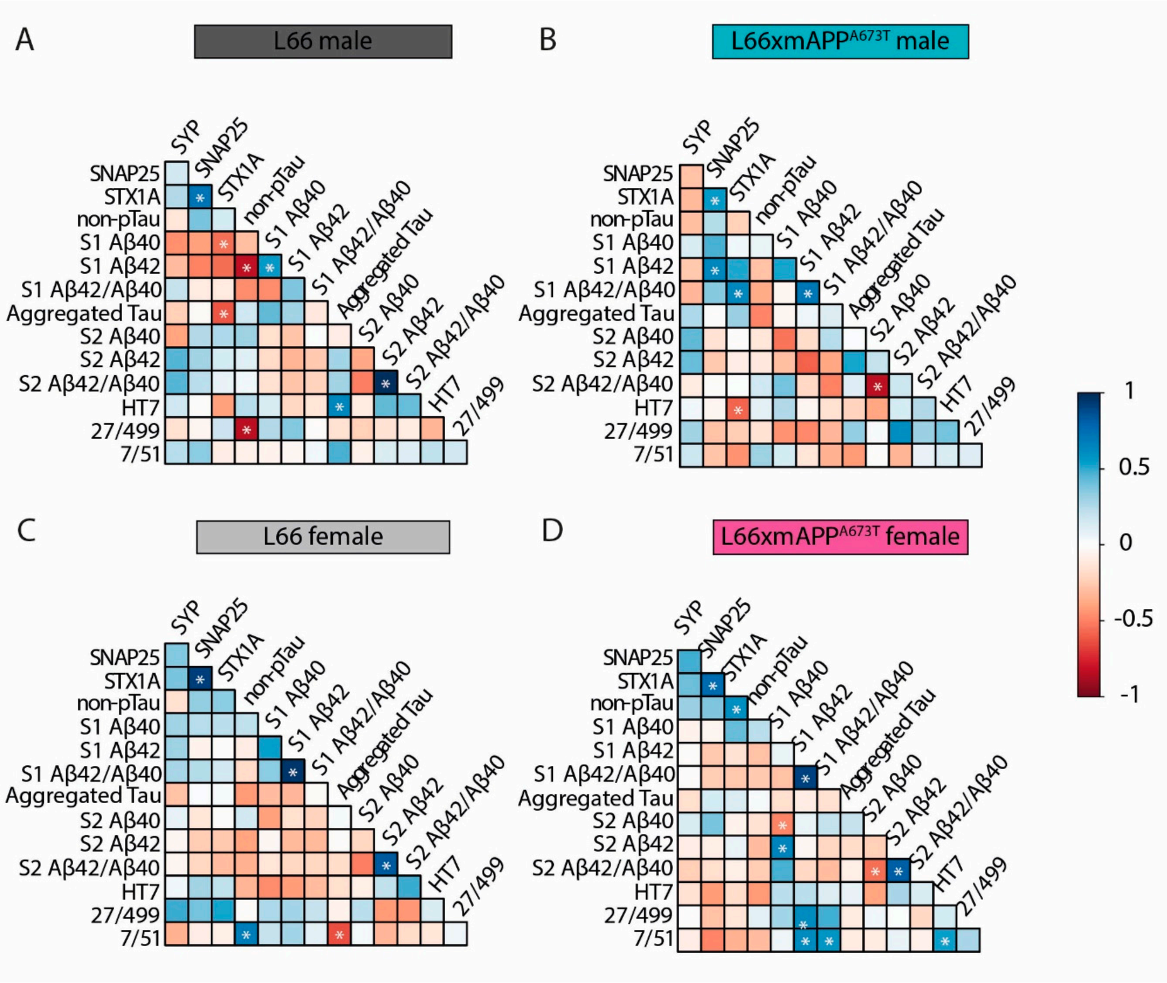

**Figure S1: Correlation matrices between tau, Aβ and synaptic proteins of L66 and L66xmAPP$^{A673T}$ mice.** Pearson correlation matrices between the synaptic proteins SYP, SNAP25, STX1A, as well as non-pTau, aggregated tau, Aβ40 and A β42 as well as their ratio are displayed for L66 male (A), L66xmA673T male (B), L66 female (C), and L66xmAPP$^{A673T}$ female (D). Blue denotes positive correlations, red for negative correlations and white indicates where no correlations were seen (* $p < 0.05$). SYP, SNAP25, STX1A, non-pTau, aggregated tau, Aβ40 and A β42 were quantified in S1 and S2 brain homogenate fractions using ELISA. HT7-tau, 27/499-tau, and 7/51-tau were quantified using immunohistochemistry (averaged across brain regions). Data were analysed using Jennrich test to detect differences between matrices. Abbreviations: SNAP25: synaptosomal associated protein 25kDa, STX1A: syntaxin 1A, SYP: synaptophysin.
